# A domain-specific annotation framework and expert-annotated corpus for structured analysis of German HNSCC pathology reports

**DOI:** 10.1186/s13104-026-08024-w

**Published:** 2026-09-21

**Authors:** Sebastian Gubik, Franziska Seger, Andreas Vollmer, Marius Hörner, Tobias Renner, Luca Kohlhepp, Marc Manthey, Urs Müller-Richter, Alexander Kübler, Stephan Hackenberg, Stefan Hartmann

**Affiliations:** 1 Department of Oral and Maxillofacial Plastic Surgery - Head and Neck surgery, University Hospital of Würzburg, Pleicherwall 2, Würzburg, Germany; 2 Department of Otorhinolaryngology, Plastic, Aesthetic, and Reconstructive Head and Neck Surgery, University Hospital Würzburg, Würzburg, Germany; 3 CAIDAS (Center of AI and Data Science), University Würzburg, Würzburg, Germany; 4 Institute of Pathology, University of Würzburg, Würzburg, Germany

**Keywords:** Clinical natural language processing, Pathology reports, Annotation schema, Medical text mining, Head and neck cancer, German medical texts, Information extraction, TNM classification

## Abstract

**Objective:**

To develop and empirically characterize a domain-specific, block-structured annotation schema and an expert-annotated reference corpus for German-language head and neck squamous cell carcinoma (HNSCC) pathology reports.

**Results description:**

We retrieved 631 pathology reports from 145 patients treated at University Hospital Würzburg between January 2021 and March 2024 and split them at the patient level into training, validation, and evaluation subsets. Reports were manually annotated using a hierarchical, span-based schema supporting overlapping and nested annotations. The resulting corpus contains 45 unique labels organized into clinically meaningful blocks and an average of 37–39 annotations per report across subsets. Label frequencies and normalized category distributions were broadly comparable across the three subsets. Fine-grained labels were predominantly applied within their designated blocks, indicating adherence to the intended block structure. The corpus provides a domain-specific reference resource for developing and evaluating structured information-extraction approaches for German HNSCC pathology reports.

**Supplementary Information:**

The online version contains supplementary material available at https://doi.org/10.1186/s13104-026-08024-w.

## Introduction

 Head and Neck Squamous Cell Carcinoma (HNSCC) ranks sixth among all cancers worldwide [1]. Besides the typical risk factors for HNSCC, tobacco and alcohol consumption, the Human Papilloma Virus (HPV) is an increasing hazard especially for younger people to be struck by HPV-associated HNSCC [2, 3]. In the last decade, a huge effort in the treatment of HNSCC was made by the introduction of check-point-inhibitor therapy. So, the general approach, which still is surgery alone, or combined with radiotherapy or radiochemotherapy [2], was complemented by new drugs like Nivolumab, Pembrolizumab or Cetuximab. Recent evidence from the phase 3 KEYNOTE-689 trial shows that perioperative pembrolizumab—administered before and after surgery—significantly improves event-free survival in patients with resectable, locally advanced HNSCC. These results underscore the growing integration of immunotherapy into curative treatment paradigms and further emphasize the importance of structured data for guiding multidisciplinary decision making as treatment options become more complex. In contrast, in the palliative setting, only approximately 10–15% of patients derive a clinically meaningful benefit from immunotherapy [4]. Since this subgroup achieves favorable clinical outcomes, it is of major interest to investigate potential markers and covariates of patients with successful, but also with negative outcome. Treatment of HNSCC is dependent on the TNM classification, by now already taking HPV status into account. The TNM classification of the tumor usually depends on the analysis of the resected tissue during operation. So, the pathology report has tremendous impact on the decision of the proceeding therapy of the patient after surgery. The pathology reports consist of semi- to unstructured text, written in German language, offering a vast amount of information. This information is clearly crucial for every single patient, but also the information which is possible to gather from multiple cases might obviously be relevant to provide more insights to cancer histology and therapy management.

In this paper, we introduce a new annotation schema designed for German-language pathology reports from patients with HNSCC and apply it to create an expert-annotated reference corpus for structured information extraction. The annotation schema was developed from a surgical perspective, with a focus on clinically relevant information that may subsequently be extracted from unstructured pathology reports and transformed into structured data for therapy evaluation and clinical research. Several approaches can be used to process free-text clinical documents, ranging from regular expressions to more sophisticated natural language processing toolkits such as the Natural Language Toolkit or spaCy [5, 6]. Large language models also offer new possibilities for clinical information extraction, although their performance in specialized clinical subdomains remains an important consideration [7, 8]. In medical applications, accurate and context-sensitive information extraction is particularly important. The annotation framework presented here provides a domain-specific basis for developing and evaluating such extraction approaches for German-language HNSCC pathology reports.

In this work, we present a domain-specific, block-structured annotation schema and an expert-annotated reference corpus for German-language pathology reports in HNSCC. The annotation schema was designed from a surgical decision-making perspective and aligns fine-grained clinical annotations with the natural structure of pathology reports. In contrast to flat annotation schemes and existing predominantly English-language clinical NLP (Natural Language Processing) resources, our approach introduces hierarchical, context-constrained labeling that supports plausibility checks across report sections and reduces ambiguity in downstream information extraction. We empirically characterize the resulting corpus by analyzing annotation density, label distributions, and annotation schema coverage across training, validation, and evaluation subsets. The presented resource establishes a methodological foundation for structured extraction of clinically relevant parameters from German HNSCC pathology reports and for future evaluation of clinical information extraction and decision-support methods.

## Materials and methods

### Corpus construction

#### Report collection—data set

To build a clinically relevant annotation corpus, we retrieved pathology reports from the Clinical Data Warehouse at the University Hospital Würzburg, Germany. The selection focused on patients diagnosed with HNSCC who underwent primary surgical treatment from January 2021 to March 2024. A total of 631 pathology reports from 145 patients were initially collected. We stratified this pool of reports by patients into three different groups:

- Training set (84 patients, 365 pathology reports) for algorithm development,
- Validation set (23 patients, 88 pathology reports) for intermediate tuning and guideline refinement,
- Evaluation set (38 patients, 178 pathology reports) as a held-out test set for final benchmarking.

Each report, written in unstructured free-text format in German, was linked to a pseudonymized patient identifier. Only reports related to the primary tumor site and lymph node assessment were included, ensuring relevance to TNM classification (TNM Classification, 8th edition, corrected reprint 2020) and postoperative treatment decisions.

#### Annotation platform and workflow

Manual annotation was performed using Label Studio (HumanSignal, San Francisco, CA, USA), an open-source annotation platform supporting span-based labeling of unstructured text. A custom labeling configuration was implemented to enable annotation using the domain-specific annotation schema, including overlapping and nested annotations required to represent the hierarchical report structure and fine-grained clinical information.

All pathology reports were annotated by a medical specialist with clinical expertise in pathology and head and neck oncology. Annotation followed a two-stage procedure. First, high-level text blocks corresponding to major report sections (e.g., submission, pathological assessment, tumor staging) were annotated to impose an explicit document structure. Second, fine-grained clinical entities were annotated within their corresponding blocks according to the annotation schema constraints. This block-constrained strategy was chosen to reflect clinical interpretation workflows and to reduce semantic ambiguity in downstream information extraction.

The annotation schema definitions and annotation guidelines were iteratively developed and refined in collaboration between the primary annotator and a second investigator with clinical and methodological expertise. The second investigator participated in the joint review of ambiguous cases and rare label occurrences, contributed to the development and refinement of annotation rules, and reviewed randomly selected annotated reports. Identified ambiguities or inconsistencies were discussed between the investigators and, where necessary, incorporated into revisions of the annotation guidelines. The second investigator did not independently annotate a predefined subset of reports; therefore, this review procedure was not designed to provide formal inter-annotator agreement estimates. Annotated data were exported in JavaScript Object Notation (JSON) format and transformed into structured representations for quantitative analysis.

### Annotation schema for the HNSCC

To standardize the annotation process and enable structured evaluation of clinical information extraction methods, we developed a domain-specific annotation schema tailored to German-language pathology reports for HNSCC. The annotation schema was designed in close collaboration with clinical experts and informed by both clinical relevance and frequency of occurrence within the corpus. Each annotation schema concept is associated with a unique label, a clear semantic definition, and an explicit clinical context.

The annotation schema follows a block-structured, hierarchical design that reflects the typical organization of pathology reports and the clinical workflow of postoperative decision-making. At the highest level, labels represent major report sections (“blocks”), such as submission information, pathological assessment, tumor staging, and resection margins. Within each block, fine-grained labels capture specific clinically relevant entities. This structure constrains the application of detailed labels to appropriate report sections, reduces semantic ambiguity, and enables plausibility checks across related components. The annotation schema and detailed annotation guidelines are publicly available to enable methodological examination and application of the proposed annotation approach to comparable datasets (https://github.com/smdg88/4paper-annotationSchema4german-HNSCC-pathology-reports).

To further characterize the semantic interoperability of the annotation schema, clinically meaningful labels were reviewed for correspondence with SNOMED CT concepts. Where an appropriate terminology concept could be identified, the annotation label was mapped to the corresponding SNOMED CT concept. Labels representing document structure, report sections, administrative information, or other task-specific contextual elements for which no semantically appropriate terminology concept was identified were designated as not applicable. The resulting mapping, including SNOMED CT concept identifiers and the mapping status of individual annotation labels, is provided in Appendix A.1, Table 6. The mapping is intended to indicate potential semantic correspondence and does not alter the original annotation schema or its block-structured organization.

#### Structural labels (report sections)

Structural labels represent high-level sections of the pathology report and serve as containers for more detailed annotations. These include, among others, report metadata (e.g., report date, sender, receiving date), descriptions of submitted specimens, pathological assessments, tumor staging statements, frozen section reports, and administrative notices such as disclaimers or correction notes. Annotating these sections explicitly allows reports to be segmented into clinically meaningful units prior to extraction of fine-grained information.

#### Labels representing tumor stage (according to AJCC/UICC)

Within tumor staging blocks, fine-grained labels capture staging information according to the AJCC/UICC TNM classification. These include labels for primary tumor extent (T), regional lymph node involvement (N), distant metastasis (M), lymphatic invasion (L), vascular invasion (V), perineural invasion (Pn), histologic grading (G), and resection status (R). Separate labels are used to distinguish preliminary, updated, and final tumor staging statements, reflecting the temporal evolution of pathological assessment.

#### Specimen submission and pathological assessment labels

Labels related to specimen submission describe how tissue samples are provided to pathology, including information on fractions or grouped specimens. Corresponding labels within pathological assessment blocks describe microscopic evaluation of submitted tissue, including localization of the primary tumor, depth of infiltration, perineural invasion, lymph node counts and levels, and detailed descriptions of tissue and lymph node findings. This parallel structure enables cross-referencing between submitted specimens and analyzed material.

#### Resection margin labels

Dedicated labels capture information on resection margins, including directional margin descriptions and distance measurements. These labels are constrained to margin-specific blocks and are critical for postoperative treatment planning and assessment of surgical completeness.

#### Design rationale and benefits

The block-structured annotation schema mirrors how clinicians read and interpret pathology reports during postoperative decision-making. By enforcing context-aware labeling, the annotation schema supports structured extraction while enabling internal consistency checks, such as verifying correspondence between submitted specimens and reported pathological findings. This design is intended to provide a structured, domain-specific basis for the development and evaluation of clinical natural language processing methods.

### Corpus limitations and annotation quality

The corpus represents an expert-annotated reference dataset created by a single clinical domain expert, with additional review by a second investigator as described in Sect.  2.1.2. The second investigator contributed to the development and refinement of the annotation guidelines, jointly reviewed ambiguous cases and rare label occurrences, and reviewed randomly selected annotated reports. However, the second investigator did not independently annotate a predefined subset of reports. Consequently, formal inter-annotator agreement metrics cannot be reported for the present corpus.

Annotation consistency was supported through iterative guideline refinement, joint review of ambiguous cases, and review of randomly selected reports. In addition, label distributions and normalized frequencies were similar across the training, validation, and evaluation subsets. These distributional findings indicate consistent use of the annotation schema across dataset subsets but should not be interpreted as a measure of annotation accuracy or inter-annotator agreement. Future work should include independent annotation by multiple annotators and formal agreement analysis, potentially involving external institutions, to quantify annotation reliability and further validate the resource.

### Materials (software)

The annotation of the data sets was performed with label studio (2025 HumanSignal, Inc.), which is an open-source labeling tool for multiple tasks, e.g. image or text annotation. The statistics was done in Python 3.12 (Wilmington, DE, USA). The coding was performed with Visual Studio Code 1.8 (Microsoft Corp., Redmond, USA).

## Results

### Annotation statistics and dataset characteristics

The annotated corpus comprises a total of 631 pathology reports from 145 patients, divided at the patient level into training (365 reports), validation (88 reports), and evaluation (178 reports) subsets. Across all subsets, reports exhibited a comparable annotation density, with an average of 37–39 annotations per document, indicating consistent annotation granularity throughout the dataset.

Table 1 summarizes the absolute label frequencies by major category across the three subsets, while Table 2 reports normalized frequencies to account for differences in subset size. The relative proportions of structural labels, submission-related labels, pathological assessment labels, margin-related labels, tumor staging labels, and other administrative labels were similar across training, validation, and evaluation datasets. These findings indicate broadly comparable label distributions across the training, validation, and evaluation subset.

**Table 1:** Label frequency by dataset

Label distribution by major category:	Training Dataset	Validation Dataset	Evaluation Dataset
Structure:	3059	742	811
Delivery Description (“Zusendung”):	1233	325	282
Tissue Analysis (“Begutachtung”):	7560	1898	1596
Margins:	332	83	117
TNM:	736	206	201
Other:	687	167	108

While some fine-grained labels occurred infrequently (e.g., M-Stadium), these were retained due to their clinical relevance and to reflect real-world reporting practices, where certain parameters are rarely documented but may be critical when present.

Detailed category-wise distribution plots for each dataset split are provided in Appendix A.2 (Figs. 1, 2, 3, 4, 5 and 6).

**Table 2:** Normalized frequencies of the labels

Normalized Frequencies (% of total annotations):	Training dataset	Validation dataset	Evaluation dataset
Structure:	22.48%	21.69%	26.04%
Delivery Description (“Zusendung”):	9.06%	9.50%	9.05%
Tissue Analysis (“Begutachtung”):	55.56%	55.48%	51.24%
Margins:	2.44%	2.43%	3.76%
TNM:	5.41%	6.02%	6.45%
Other:	5.05%	4.88%	3.47%

### Annotation schema coverage and structural consistency

The annotation schema comprises 45 unique annotation labels organized into clinically meaningful blocks corresponding to major sections of pathology reports. Figure A7 illustrates the coverage of the annotation schema across these blocks for the complete dataset. All blocks were consistently populated, with the majority of annotations occurring within pathological assessment and structural sections, reflecting the detailed descriptive nature of pathology reports.

To assess whether fine-grained labels were applied in accordance with the intended block structure, we analyzed label usage across blocks. The resulting heatmap (Figure A8) demonstrates that detailed clinical labels were almost exclusively used within their designated blocks, indicating adherence to the intended context constraints across the corpus.

Labels that appeared exclusively in the training set but not in validation or evaluation subsets were identified (e.g., M-Stadium, Befundkorrekturhinweis). These cases reflect the natural sparsity of certain clinically relevant events rather than inconsistencies in annotation practice.

### Annotation example

An example of a fully annotated pathology report, illustrating the hierarchical block structure and overlapping span annotations, is provided in Appendix A.2 (Fig. 9a-c). This example demonstrates how high-level report sections were annotated prior to labeling fine-grained clinical entities within their corresponding blocks.

## Discussion

### Significance of the corpus and design rationale

In this paper, we present a domain-specific, German-language annotated corpus for HNSCC pathology reports from the perspective of head and neck surgery. This work addresses a critical gap in German clinical natural language processing (NLP), as most existing annotated corpora are available only in the English language [8]. Moreover, it provides a foundation for both medical research and routine clinical application. For research purposes, the dataset enables structured extraction of clinically relevant parameters and facilitates the identification of potential associations and correlations across cases [5, 6, 9, 10]. In addition, the corpus supports methodologically grounded evaluation of information extraction (IE) approaches in this specialized domain [11, 12]. Such approaches can be applied both in research settings and in clinical routine, where automated extraction and presentation of annotation schema defined parameters may help reduce physicians’ daily workload and support informed decision-making. Existing biomedical ontologies such as SNOMED CT and the Unified Medical Language System (UMLS) provide extensive controlled vocabularies for clinical concepts. However, the present work focuses on a pragmatic annotation schema designed specifically for span-based labeling and structured information extraction from German-language pathology reports rather than on terminology-based normalization of extracted clinical concepts. The framework emphasizes document structure and clinically relevant entities that are relevant for surgical decision-making and information extraction from real-world pathology narratives. A direct one-to-one mapping of the complete annotation schema to established clinical terminologies such as SNOMED CT is not feasible because the two approaches serve different representational purposes. While some fine-grained labels describe clinical concepts for which corresponding standardized concepts may exist, a substantial part of the proposed schema represents document structure, contextual information, or relationships between sections of the pathology report rather than standalone clinical concepts. These elements are essential for the block-structured information extraction approach presented here but are not necessarily represented as equivalent concepts in clinical terminologies. Consequently, assigning terminology concepts to all labels solely to establish formal correspondence could introduce artificial or semantically imprecise mappings.

Nevertheless, semantic interoperability remains an important consideration for downstream applications of the corpus. To assess the relationship between the proposed annotation schema and standardized clinical terminology, we reviewed the clinically meaningful annotation labels for correspondence with SNOMED CT concepts (Appendix A.1, Table 6). Where a semantically appropriate correspondence could be identified, the annotation label was mapped to the respective SNOMED CT concept. Structural, administrative, and task-specific contextual labels for which no appropriate terminology correspondence was identified were designated as not applicable. This partial mapping illustrates that standardized terminology can complement the block-structured annotation framework without requiring all document-level labels to be represented as standalone terminology concepts. For downstream applications, extracted clinical information may therefore be linked to standardized terminology where appropriate, while the original annotation structure preserves the contextual organization required for the information extraction task.

The annotation schema is organized into thematic blocks (e.g., “Zusendung”, “Begutachtung”, “Tumorstadium”) to enable an initial structural segmentation of pathology reports. For annotators, this block-based design provides a clinically intuitive framework for labeling, closely reflecting how pathology reports are read and interpreted in routine practice.

Beyond annotation, the block structure offers clear advantages for downstream algorithm design. Information extraction algorithms can leverage the predefined structure to reduce computational complexity, as not all labels need to be evaluated across the entire document. Instead, documents are segmented into clinically relevant blocks, and fine-grained sub-labels are extracted only within their corresponding sections. This targeted extraction strategy can improve efficiency and reduce false-positive detections.

In addition, the block-based annotation schema enables internal cross-referencing and plausibility checks. For example, correspondence between the “Zusendung” and “Begutachtung” blocks allows verification that the number of tissue fractions analyzed pathologically matches the number of specimens documented as received from surgery. While the “Zusendung” block records which tissue samples were submitted and accepted by pathology, the “Begutachtung” block contains the corresponding microscopic analyses. Such cross-block consistency checks may help identify inconsistent extracted results and can be explicitly integrated into information extraction pipelines.

Finally, analysis of annotation schema usage statistics showed that labels were predominantly applied within their designated blocks. Label frequencies and coverage were similar across training, validation, and evaluation datasets, indicating comparable use of the annotation schema across the three dataset subsets.

Annotation of German pathology reports is challenging because of linguistic variability, heterogeneous reporting practices, and evolving diagnostic interpretations. The annotation schema was designed to accommodate these sources of variation while preserving clinically relevant information.

### Limitations

Manual annotation of a specialized clinical corpus is inherently time-consuming and resource-intensive, particularly given the limited availability of clinicians actively involved in routine patient care. The overall dataset size (631 pathology reports) may appear modest; however, it reflects the highly specialized nature of HNSCC pathology and the strict inclusion criteria applied in this study. Furthermore, the dataset originates from a single institution, the University Hospital Würzburg, which may limit generalizability to other clinical settings. Nevertheless, Würzburg is a dedicated tumor center with a high annual case volume, and reporting practices are representative of standard care in German university hospitals.

The annotation schema was developed specifically for document-level annotation and information extraction rather than being derived from an established clinical terminology. This task-specific design limits direct semantic interoperability with ontology-based datasets. Because the schema includes both clinical concepts and document-structural or contextual labels, a complete one-to-one mapping to terminologies such as SNOMED CT is not semantically appropriate. To improve semantic interoperability, clinically meaningful labels were mapped to corresponding SNOMED CT concepts where an appropriate correspondence could be identified (Appendix A.1, Table 6), while structural and task-specific contextual labels without an appropriate terminology correspondence were designated as not applicable. This mapping is limited to semantic correspondence at the annotation-schema level and does not constitute a complete terminology-normalization framework for extracted clinical information. Future work should evaluate terminology normalization of extracted values in downstream applications and assess its applicability across institutions and reporting practices.

A further limitation is that annotation was performed primarily by a single expert, with a second investigator reviewing ambiguous cases and randomly selected annotated reports and contributing to the development and refinement of the annotation rules. While this review procedure was intended to support consistent application of the annotation schema, the second investigator did not independently annotate a predefined subset of reports. Consequently, formal inter-annotator agreement could not be assessed. Future studies should include independent annotation by multiple annotators and formal inter-annotator agreement analysis to quantify annotation reliability and further validate the corpus.

## Conclusion

In this study, we developed and empirically characterized a domain-specific, block-structured annotation schema and an expert-annotated reference corpus for German-language pathology reports in head and neck squamous cell carcinoma. By aligning fine-grained clinical annotations with the natural structure of pathology reports, the proposed approach enables structured extraction of clinically relevant parameters while preserving contextual integrity.

The presented work addresses a gap in German clinical natural language processing and provides a domain-specific methodological basis for the development and evaluation of information extraction approaches in a specialized clinical setting. The underlying pathology reports and annotated corpus cannot be made publicly available because of legal and ethical restrictions related to patient privacy and data protection. However, the annotation schema, label definitions, and annotation guidelines are publicly available in an online repository (https://github.com/smdg88/4paper-annotationSchema4german-HNSCC-pathology-reports), enabling the proposed annotation methodology to be examined and applied to comparable institutional datasets. Future studies should evaluate information extraction approaches using the presented framework and perform independent multi-annotator assessment to quantify annotation reliability.

## Supplementary Information

Below is the link to the electronic supplementary material.

Supplementary Material 1

## Data Availability

No datasets were generated or analysed during the current study.

## Abbreviations

HNSCC: Head and Neck Squamous Cell Carcinoma
TNM: Tumor, Node, Metastasis
HPV: Human Papilloma Virus
NLP: Natural Language Processing
IE: Information Extraction
IRB: Institutional Review Board
UMLS: Unified Medical Language System
GDPR: General Data Protection Regulation
