# Supplementary material for: A domain-specific annotation framework and expert-annotated corpus for structured analysis of German HNSCC pathology reports
Source: BMC Res Notes. 2026 Sep 21;19:355. doi: 10.1186/s13104-026-08024-w (PMC13595725; doi:10.1186/s13104-026-08024-w)
Supplement: Supplementary file 1 [file 13104_2026_8024_MOESM1_ESM.docx]

**Appendix A:**

A.1 Tables:

Table 1: Labels representing text-structure.

| Label (in German) | Information |
| --- | --- |
| Pathobefund-Datum | Representing the date of the report. |
| Einsender | Representing the clinic sending the pathology request. |
| Eingangsdatum | Representing the date/time of receiving the preparation (for sorting). |
| Operateur | Representing the operating surgeon. |
| Zusendung | Representing the Description of the incoming preparation. |
| Abschließende Begutachtung | Representing the final analysis of the tissue. |
| Vorläufige Begutachtung | Representing the  preliminary analysis of the tissue |
| Begutachtung | Representing the analysis of the tissue. |
| Vorläufiges Tumorstadium | Representing the  preliminary tumor stage of the tissue (e.g. necessary when bone tissue is not yet finally analyzed) |
| Abschließendes Tumorstadium | Representing the final tumor stage of the tissue. |
| Aktualisiertes Tumorstadium | Representing the updated tumor stage of the tissue. |
| Tumorstadium | Representing the tumor stage without specification. |
| Abstand | Representing the section, where the margins are shown. |
| Schnellschnitt (Telefon) | Representing the “Frozen Section” information (often made via telephone directly to the OR) |
| Schnellschnitt (Detail) | Representing the “Frozen Section” information (with detailed information) |
| Befunder | Representing the reporting pathologist. |
| Disclaimer | A disclaimer often shown at the end of the reports. |
| Befundkorrekturhinweis | Representing a notice (e.g. correction notice – wrong tumor stadium) |

Table 2: Labels representing Tumor Staging.

| Label | Information |
| --- | --- |
| T-Stadium | Representing the primary tumor (e.g. T1-T4) |
| N-Stadium | Representing the lymph node metastasis (e.g. N0-N3) |
| M-Stadium | Representing distant Metastasis (e.g. Mx, M0, M1) |
| L-Stadium | Representing Lymphatic Invasion (e.g. L0, L1) |
| V-Stadium | Representing Vascular Invasion (e.g. V0, V1, V2) |
| Pn-Stadium | Representing Perineural Invasion (e.g. Pn0, Pn1) |
| Grading | Representing the histologic grade (e.g. G1, G2, G3, G4) |
| R-Stadium | Representing the Resection Status (e.g. R0, R1, R2) |

Table 3: Labels representing relevant parts of the submitted preparation.

| Label | Information |
| --- | --- |
| Fraktion-Zusendung | Representing the Fractions in which different preparations are sent to the pathologist. |

Table 4: Labels representing relevant parts of the pathology analysis of the tissue.

| Label | Information |
| --- | --- |
| Fraktion-Begutachtung | Representing the fractions of tissue samples analyzed by the pathologist. |
| Primarius | Representing the description of the localization of the primary tumor. |
| Infiltrationstiefe (Primarius) | Representing the depth of Infiltration of the primary tumor. |
| Perineuralscheideninfiltration | Representing the infiltration of the tumor into neural tissue. |
| LK-Anzahl | Representing the count of extracted and affected lymph nodes (according to the fraction and level) |
| LK-Level | Representing the anatomical lymph node level (e.g., Level I–V). |
| Lymphknoten-Begutachtung | Representing the pathological analysis of the lymph node tissue (according to the fraction and level) |
| Gewebe-Begutachtung | Representing the pathological analysis of the extracted tissue (according to the fraction and level) |

Table 5: Labels representing the margins of the tumor preparation.

| Label | Information |
| --- | --- |
| Abstand Richtung | Representing the direction and the margins of the resection of the primary tumor preparation. |

Table 6: Mapping of annotation-schema labels to standardized clinical terminology.

| Label | Meaning in this schema | Type | Proposed standardized concept / terminology | Identifier | Mapping status / rationale |
| --- | --- | --- | --- | --- | --- |
| Pathobefund-Datum | Date of pathology report | Structural/  metadata | N/A | N/A | Document metadata; no clinical concept mapping required |
| Einsender | Submitting clinic or department | Structural/  metadata | N/A | N/A | Administrative/  document-context label |
| Eingangs-datum | Date/time specimen received | Structural/  metadata | N/A | N/A | Workflow/ document metadata |
| Operateur | Operating surgeon | Structural/  metadata | N/A | N/A | Person/role information rather than clinical finding |
| Befunder | Reporting pathologist | Structural/  metadata | N/A | N/A | Person/role information rather than clinical finding |
| Disclaimer | Administrative/  legal disclaimer | Administrative | N/A | N/A | Document-structural/  administrative label |
| Befund-korrektur-hinweis | Correction/  amendment notice | Administrative | N/A | N/A | Document-status/context label |
| Zusendung | Narrative description of submitted specimens | Structural | N/A | N/A | Report block; individual specimen content could be normalized downstream |
| Fraktion-Zusendung | Individual submitted specimen fraction | Structural/  contextual | N/A | N/A | Task-specific fraction identifier/  description; value-dependent specimen/  anatomical normalization possible downstream |
| Begutachtung | Pathological assessment | Structural | N/A | N/A | Report block rather than standalone clinical concept |
| Aktualisierte Begutachtung | Updated pathological assessment | Structural/  contextual | N/A | N/A | Report block with temporal/  document-status context |
| Abschließende Begutachtung | Final pathological assessment | Structural/contextual | N/A | N/A | Report block with finality context |
| Vorläufige Begutachtung | Preliminary pathological assessment | Structural/  contextual | N/A | N/A | Report block with preliminary-status context |
| Fraktion-Begutachtung | Assessment of a specific specimen fraction | Structural/  contextual | N/A | N/A | Links findings to a task-specific specimen fraction |
| Gewebe-Begutachtung | Tissue-specific pathological findings | Clinical/  contextual | No single fixed concept | — | Broad span may contain multiple histological findings; terminology mapping is value-dependent |
| Lymphknoten-Begutachtung | Pathological findings of lymph-node tissue | Clinical/  contextual | Presence of metastatic neoplasm in lymph node in excised tissue specimen | 1268302004 | Related SNOMED CT observable available; annotated span may contain broader findings, so not an exact one-to-one mapping |
| LK-Anzahl | Number of examined and/  or affected lymph nodes | Clinical | Number of lymph nodes examined by microscopy in excised specimen; Number of lymph nodes containing metastatic neoplasm in excised specimen | 444025001; 443527007 | Value/meaning dependent: schema label combines examined and/or involved counts; two separate SNOMED CT observables exist |
| LK-Level | Anatomical lymph-node level | Clinical | Anatomic location of excised lymph node containing metastatic neoplasm | 397440000 | Related observable; actual lymph-node level/  anatomical structure is value-dependent |
| Primarius | Localization of primary tumor | Clinical | Primary tumor site/ anatomic location of primary malignant neoplasm | 399687005 | Close schema-level correspondence; actual anatomical site is encoded by the annotated value |
| Infiltrations-tiefe (Primarius) | Depth of primary-tumor infiltration | Clinical | SNOMED CT cancer synoptic invasion/  extension observable model | — | Value/site dependent; no single HNSCC-independent concept should be imposed without specifying tumor site and measurement context |
| Perineural-scheiden-infiltration | Tumor infiltration of nerve sheaths | Clinical | Status of perineural invasion by tumor / presence of direct invasion by primary malignant neoplasm to nerve | 371513001 | Close correspondence; observation value determines presence/absence |
| Tumorstadium | Unspecified/  preliminary TNM staging statement | Structural/  contextual | UICC TNM / SNOMED CT UICC staging content | — | Block containing staging information; map individual TNM values rather than the block itself |
| Aktualisiertes Tumorstadium | Updated TNM staging | Structural/  contextual | UICC TNM / SNOMED CT UICC staging content | — | Temporal/  document-status block; individual TNM values are mapped |
| Abschließendes Tumorstadium | Final TNM staging | Structural/  contextual | UICC TNM / SNOMED CT UICC staging content | — | Finality is annotation context; individual TNM values are mapped |
| Vorläufiges Tumorstadium | Preliminary TNM staging | Structural/  contextual | UICC TNM / SNOMED CT UICC staging content | — | Preliminary status is annotation context; individual TNM values are mapped |
| T-Stadium | Primary tumor extent;  T-category expression | Clinical/staging | UICC TNM categories represented in SNOMED CT | UICC/SNOMED value-dependent | Value-dependent mapping (e.g., pT1, pT2, etc.); no single SCTID represents all annotated T values |
| N-Stadium | Regional lymph-node involvement; N-category expression | Clinical/staging | UICC TNM categories represented in SNOMED CT | UICC/SNOMED value-dependent | Value-dependent mapping (e.g., pN0, pN1, etc.) |
| M-Stadium | Presence/  absence of distant metastasis; M-category expression | Clinical/staging | UICC TNM categories represented in SNOMED CT | UICC/SNOMED value-dependent | Value-dependent mapping (e.g., cM0, pM1, etc.) |
| L-Stadium | Lymphatic invasion status; L-category | Clinical/staging | Status of lymphatic (small vessel) invasion by tumor | 395715009 | Close correspondence; actual L category/value should be represented separately |
| V-Stadium | Vascular/  venous invasion status; V-category | Clinical/staging | Status of venous (large vessel) invasion by tumor | 371493002 | Close correspondence for UICC V category; value-dependent |
| Pn-Stadium | Perineural invasion status; Pn-category | Clinical/staging | Status of perineural invasion by tumor / presence of direct invasion by primary malignant neoplasm to nerve | 371513001 | Close correspondence; Pn value remains value-dependent |
| Grading | Histologic tumor grade; G-category | Clinical | Histologic grade (observable entity) | 371469007 | General schema-level correspondence; site-/method-specific SNOMED CT observables may provide more precise representation |
| R-Stadium | Resection/  residual-tumor status; R-category | Clinical/  staging | Residual tumor stage | 17964000 | Close staging-level correspondence; actual R category is value-dependent |
| Abstand | Section describing resection margins | Structural | N/A | N/A | Margin-specific report block, not itself a clinical observation |
| Abstand Richtung | Direction-specific margin/  distance information | Clinical/  contextual | Distance of neoplasm from closest surgical margin in excised specimen | 371489008 | Related/close mapping for distance; direction and anatomical margin remain value-dependent |
| Schnellschnitt (Telefon) | Frozen-section information communicated by telephone | Structural/  contextual | N/A | N/A | Report block and communication modality rather than a specific clinical finding |
| Schnellschnitt (Detail) | Detailed frozen-section report | Structural/  contextual | N/A | N/A | Report block; individual findings can be normalized downstream |

The mapping represents semantic correspondence at the annotation-schema level and does not imply exact equivalence between every annotation label and a single SNOMED CT concept. For labels capturing variable clinical values, such as TNM categories, anatomical locations, lymph-node counts, and margin distances, the appropriate terminology representation depends on the annotated value and clinical context. Structural, administrative, and task-specific contextual labels were designated N/A where terminology mapping would not be semantically appropriate.

A.2 Figures:

Figure 1: Label Frequency per Category - Training Dataset.

Figure 2: Label Frequency per Category - Validation Dataset.

Figure 3: Label Frequency per Category - Evaluation Dataset.

Figure 4: Label Category Distribution – Train Data Set.

Figure 5: Label Category Distribution – Validation Data Set.

Figure 6: Label Category Distribution – Evaluation Data Set

Figure 7: The annotation schema coverage of the complete dataset per block.

*Figure 8: Heatmap for Illustrating the Usage of the Lables per Block.*

Figure 9a: Example Representation of an Annotated Document.

Figure 9b: Example Representation of an Annotated Document.

Figure 9c: Example Representation of an Annotated Document.
